# Comprehensive Analysis of Exosomal microRNAs in Buffalo Milk Across the Early Postpartum Transition

**DOI:** 10.3390/molecules31081332

**Published:** 2026-04-18

**Authors:** Jiazheng Zhu, Rongchun Huang, Pingbai Liu, Yuan Yang, Yue Zhang, Shengfei Yan, Gan Liang, Meiting Chen, Mengyuan Zhou, Guangsheng Qin, Qiang Fu

**Affiliations:** 1 College of Animal Science and Technology, Guangxi University, Nanning 530004, China; ggbond0330@163.com (J.Z.); 18386484298@163.com (P.L.); 18211816773@163.com (Y.Y.); zhangyue200002@163.com (Y.Z.); 2 Buffalo Research Institute, Chinese Academy of Agricultural Sciences, Nanning 530001, China; 18977545979@163.com (R.H.); yanshengfei2020@163.com (S.Y.); 18776452585@163.com (G.L.); 2112159161@stu.fosu.edu.cn (M.C.); 18900296501@163.com (M.Z.)

**Keywords:** milk-derived exosomes, lactation stages, microRNAs (miRNAs), macrophage, immunomodulation

## Abstract

Milk-derived exosomes (MDEs) are bioactive nanocarriers rich in microRNAs (miRNAs) that play critical roles in post-transcriptional regulation during neonatal development and immune adaptation. However, the dynamic changes in miRNA expression across lactation stages and their biological functions remain insufficiently explored. We hypothesized that the miRNA cargo of buffalo MDEs exhibits temporal specificity, thereby dynamically matching the immune requirements of the neonatal calves. Therefore, the present study aimed to systematically characterize the miRNA expression profiles of MDEs derived from colostrum, transitional milk, and mature milk. MDEs were isolated, purified using differential ultracentrifugation, and characterized via transmission electron microscopy, Western blotting, and nanoparticle-tracking analysis. A total of 370 miRNAs were identified in the MDEs, with 220 (59.5%) co-expressed across colostrum, transitional milk, and mature milk. Comparative analysis revealed that colostrum MDEs exhibited the greatest miRNA diversity. Expression patterns of miRNAs showed distinct stage-specific clustering as lactation progressed. Compared to mature milk, 100 differentially expressed miRNAs (DE-miRNAs) were identified in colostrum MDEs, including 39 upregulated and 61 downregulated miRNAs. Bioinformatics analyses indicated that predicted target genes were associated with transmembrane transport, immune response, cell development, and apoptosis. Kyoto Encyclopedia of Genes and Genomes (KEGG) pathway enrichment analysis identified pathways involved in immune regulation, inflammation, and apoptosis. Moreover, macrophages incubated with buffalo colostrum MDEs showed upregulation of proliferation-related genes and downregulation of pro-inflammatory factors, suggesting an anti-inflammatory effect through activation of the phosphoinositide 3-kinase-protein kinase B (PI3K-Akt) signaling pathway. These findings offer new insights into miRNA profiles of buffalo MDEs across the early postpartum transition and provide a preliminary basis for exploring immunomodulatory potential of buffalo MDEs.

## 1. Introduction

Water buffalo rank as the second-largest producers of milk among livestock species [1]. While mature buffalo milk is primarily recognized for its high lipid, protein, and lactose content to support the rapid metabolic and structural growth of the neonate [2], buffalo colostrum represents a distinct, transient biofluid secreted immediately postpartum. Neonatal ruminants are born with a naive immune system due to the placental barrier, which prevents the in utero transfer of maternal macromolecules [3]. Consequently, the survival and immunological development of the developing neonatal organism depend exclusively on the acquisition of passive immunity through the ingestion of colostrum. Colostrum is chemically distinct from mature milk, as it is highly enriched in immunoglobulins (IgG), lactoferrin, maternal leukocytes, and cytokines [4]. As the neonatal immune system matures, the composition of milk dynamically shifts to mature milk, with its primary biological function from immune protection to nutritional homeostasis.

In addition to basic nutrients, milk-derived exosomes (MDEs) are recognized as bioactive nanocarriers capable of delivering molecular signals [5]. These nanoscale vesicles, ranging from 30 to 150 nm in size, are enclosed by a phospholipid bilayer and are distinguished by their unique structure and physiological functions [6]. MDEs exhibit remarkable stability, resisting degradation by gastrointestinal enzymes and acidic environments, which facilitates the targeted delivery of cargo to intestinal cells [7]. Proteomic analyses suggest that MDEs may play significant roles in immunomodulation and metabolic regulation [8]. Among their cargo, microRNAs (miRNAs) exert post-transcriptional regulatory effects on target gene expression [9]. Notably, milk-derived miRNAs can cross species barriers and influence gene regulation in recipient organisms [5,10].

Upon oral administration, MDEs interact with the intestinal mucosa, where intestinal macrophages act as primary target cells responsible for maintaining mucosal homeostasis and defending against pathogens [11,12]. Emerging evidence indicates that MDEs can alleviate intestinal inflammation and tissue injury by modulating macrophage polarization [13]. Specific miRNAs within MDEs, such as *let-7c*, can modulate the PTEN-PI3K/Akt signaling axis or inhibit pro-inflammatory factor release via the Toll-like receptor 4/nuclear factor kappa B (TLR4/NF-κB) pathway [14,15]. Other MDE miRNAs, such as *miR-27b*, can induce endoplasmic reticulum stress, influencing cell fate decisions [16].

Lactation is a dynamic process, during which milk composition changes over time to meet the immunological needs of the offspring [17]. A recent comparative study of colostrum and mature milk MDEs from Holstein and Jersey cows revealed significant differences in miRNA profiles, primarily associated with immune-related pathways [18]. However, variations in miRNA profiles during the transition from colostrum to mature milk remain underexplored. MDEs serve as pivotal mediators of maternal–neonatal communication. Temporal changes in MDE miRNA profiles from colostrum to mature milk are hypothesized to reflect the neonate’s shifting physiological needs. Despite their critical functions, the dynamic variations in buffalo MDE miRNA profiles across the lactational transition remain underexplored. Characterizing MDE miRNAs could provide insights into their role in enhancing neonatal immune development. The present study aimed to systematically profile and compare the miRNA cargo of buffalo MDEs from colostrum, transitional milk, and mature milk (Day 10). We hypothesized that colostrum MDEs are specifically enriched in immune-related miRNAs to facilitate neonatal immune maturation. This study provides a preliminary theoretical foundation for elucidating the immunomodulatory roles and biological significance of buffalo MDEs in supporting neonatal immune maturation.

## 2. Results

### 2.1. Morphology of Exosomes

**Figure 1:**
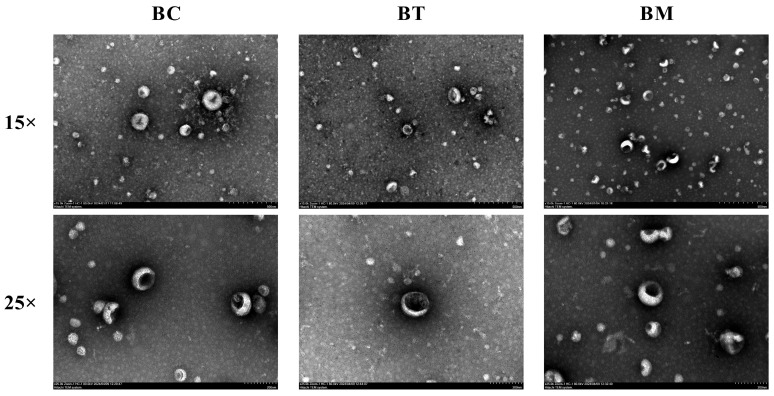
TEM images of buffalo MDEs at different lactation stages. BC, BT, and BM represent colostrum, transitional milk, and mature milk, respectively. Scale bar = 500 nm.

As shown in Figure 1, the isolated vesicles displayed a characteristic cup-shaped or saucer-like structure, with intact membranes and a clearly defined bilayer, a hallmark of exosomes. No significant morphological differences were observed among MDEs from different lactation stages. These morphological results provide initial evidence for the successful isolation of exosome-like vesicles from the complex matrix of buffalo milk.

### 2.2. Characterization of MDEs

**Figure 2:**
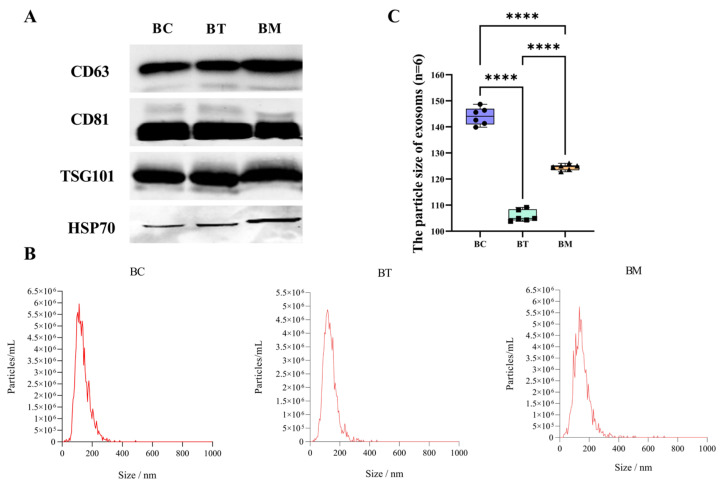
Characterization of buffalo MDEs. (**A**) Western blot analysis of exosomal markers. (**B**) Particle size distribution by NTA. (**C**) Box-and-whisker plot illustrating the particle size distribution of buffalo MDEs across lactation stages. The purple, light green, and orange boxes represent BC, BT, and BM, respectively. Superimposed individual data points represent distinct biological replicates (*n* = 6 lactating buffaloes per group). BC, BT, and BM indicate colostrum, transitional milk, and mature milk, respectively. **** *p* < 0.0001.

Western blotting analysis was performed to detect three classical exosomal markers. As shown in Figure 2A, high levels of TSG101, CD63, and CD81 were detected in all samples, and HSP70 was also positively expressed. Figure 2B shows that the average diameters of exosomes from colostrum, transitional milk, and mature milk were 148.7 nm, 104.3 nm, and 125.0 nm, respectively. The particle sizes of all samples primarily ranged from 30–200 nm, in line with the MISEV2023 guidelines from the International Society for Extracellular Vesicles [19]. Statistical analysis of the individual biological replicates (Figure 2C) revealed significant stage-specific variance in exosomal particle size, with colostrum-derived MDEs exhibiting the largest diameter and the broadest size distribution among three lactation stages. These results demonstrate that the exosomes were successfully isolated from the complex matrix of buffalo milk.

### 2.3. miRNAs Profiles of MDEs

**Figure 3:**
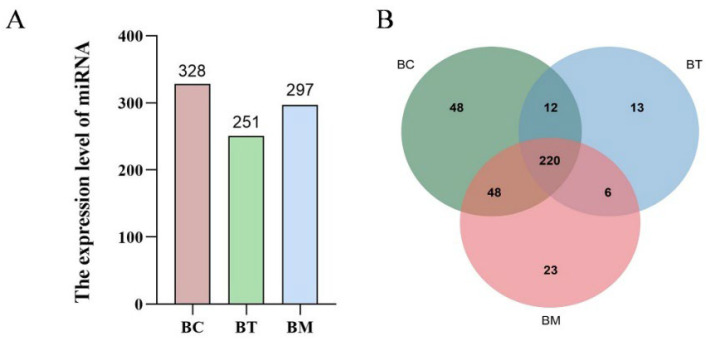
miRNA profiles of MDEs from different lactation stages. BC, BT, and BM represent colostrum, transitional milk, and mature milk, respectively. (**A**) Number of miRNAs across different lactation stages. (**B**) Venn diagram of miRNA distribution.

MDEs from buffalo colostrum, transitional milk, and mature milk underwent miRNA sequencing. A total of 13,006,482 to 15,347,736 clean reads were obtained from the miRNA libraries of the three groups. All Q30 values exceeded 97%, and GC content ranged from 45.0% to 48.0%, indicating high sequencing quality. The numbers of reads mapping to the buffalo genome were 9,529,106, 5,712,656, and 11,642,449 for colostrum, transitional milk, and mature milk, respectively. Mapping statistics are summarized in Table 1. A total of 370 miRNAs were identified in the MDEs, with 328, 251, and 297 miRNAs identified from colostrum, transitional milk, and mature milk, respectively. Colostrum MDEs contained the highest number of miRNAs (Figure 3A). Venn analysis revealed that 220 (59.5%) miRNAs were co-expressed across all three lactation stages, while 48 (13.0%), 13 (3.5%), and 23 (6.2%) miRNAs were uniquely expressed in colostrum, transitional milk, and mature milk, respectively (Figure 3B).

The top 10 most highly expressed miRNAs across all three groups accounted for 89.1% to 90.0% of the total clean reads and were conserved across multiple species (Table S1). These abundantly expressed miRNAs belonged to six miRNA families, including *miR-let-7* (*let-7a-5p*, *let-7b*, *let-7f*, *let-7g*, *let-7c*), *miR-26a*, *miR-30a-5p*, *miR-16a*, and *miR-191*, *bta-miR-200c*.

### 2.4. Differentially Expressed miRNAs in MDEs

**Figure 4:**
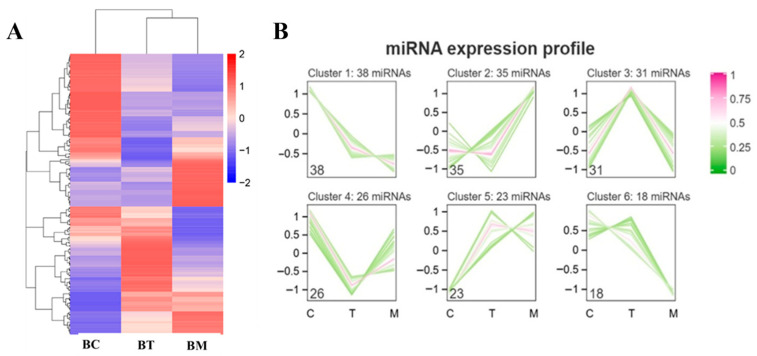
Hierarchical clustering of DE-miRNAs. (**A**) Heatmap of miRNA expression; (**B**) Cluster analysis of DE-miRNAs. BC, BT, and BM represent colostrum, transitional milk, and mature milk, respectively. Red indicates upregulation, green indicates downregulation.

As shown in Figure 4A, a heatmap visualized distinct miRNA expression patterns across the lactation stages. In total, 171 differentially expressed miRNAs (DE-miRNAs) were identified, which clustered into six expression patterns (Figure 4B). Among these, 38 miRNAs exhibited a gradual decrease from colostrum to mature milk, 35 miRNAs remained stable between colostrum and transitional milk but increased in mature milk, and 31 miRNAs were highly expressed in transitional milk. Compared to mature milk, 100 DE-miRNAs were identified in colostrum MDEs, comprising 39 upregulated and 61 downregulated miRNAs, while 52 DE-miRNAs were identified in transitional milk, with 24 upregulated and 28 downregulated miRNAs (Table S2). These results demonstrate dynamic changes in MDE miRNA profiles during lactation.

### 2.5. Functional Annotation of the Predicted miRNA Target Genes

**Figure 5:**
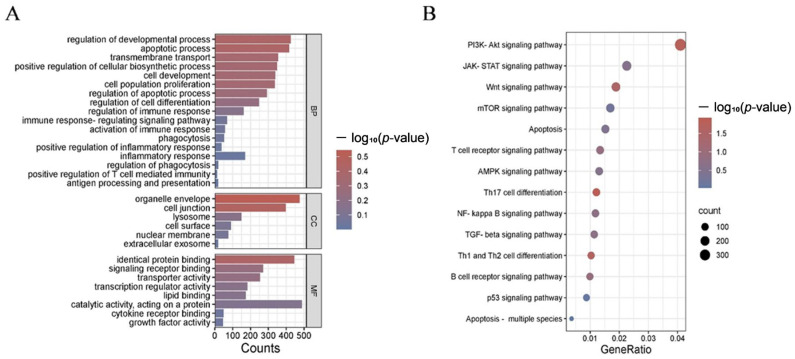
Functional enrichment analysis of predicted target genes of DE-miRNAs. (**A**) GO annotation; (**B**) KEGG pathway enrichment.

miRanda was employed to predict target genes, using the thresholds of Energy < −10 kcal/mol and alignment length ≥ 8. Gene Ontology (GO) annotation revealed that the predicted target genes were primarily enriched in biological processes such as transmembrane transport, immune response, cell development, and apoptosis. Regarding cellular components, the target genes were mainly associated with cell junctions, lysosomes, organelle membranes, cell surfaces, and nuclear membranes. In terms of molecular function, the target genes were involved in signaling receptor binding, transcription regulation, growth factor activity, catalytic activity, and transporter activity (Figure 5A). KEGG pathway analysis indicated that enriched pathways were mainly involved in immune regulation, inflammatory response, and apoptosis, including the AMP-activated protein kinase (AMPK), Janus kinase-signal transducer and activator of transcription (JAK-STAT), PI3K-Akt, and apoptosis pathways (Figure 5B) (Table S3).

### 2.6. Integrated miRNA-mRNA Network Analysis

**Figure 6:**
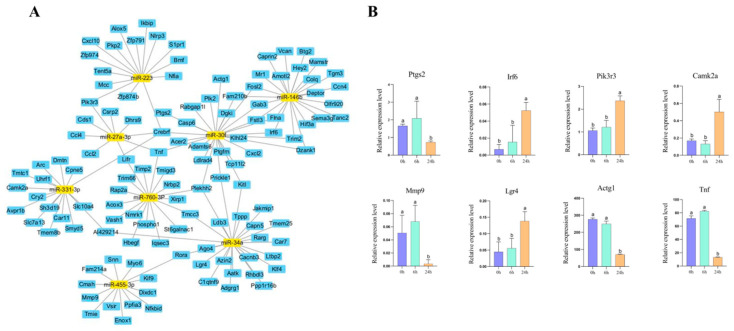
Integrated analysis of DE-miRNAs and target genes. (**A**) Interaction network of DE-miRNAs and mRNAs. The yellow nodes represent miRNAs, and the blue nodes represent target genes. (**B**) Expression levels of key genes in signaling pathways in macrophages. The purple, light green, and orange bars represent 0 h, 6 h, and 24 h of MDE incubation, respectively. Data are presented as mean ± SD. Different letters indicate significant differences between groups (*p* < 0.05).

The predicted target genes associated with immune-related pathways were retrieved and visualized using Cytoscape software (version 3.10.1). The miRNA-mRNA interaction network identified several hub miRNAs, including *miR-223*, *miR-146b*, *miR-27a-3p*, *miR-30f*, *miR-331-3p*, *miR-760-3p*, *miR-34a*, and *miR-455-3p*, as key regulators of immunomodulation (Figure 6A). In macrophages, genes related to proliferation, such as *Irf6*, *Camk2a*, and *Pik3r3*, were significantly upregulated after 24 h of MDE incubation, while inflammation-related genes, including *Ptgs2*, *Mmp9*, and *Tnf*, were downregulated (Figure 6B). These results indicate that buffalo MDEs modulate macrophage function through specific miRNA-mRNA interactions, primarily activating the PI3K-Akt signaling pathway and suppressing the inflammatory response.

## 3. Discussion

MDEs are recognized as transporters of miRNAs that regulate gene expression in target cells [7,20]. However, a comprehensive analysis of buffalo MDEs remains limited. This study hypothesizes that buffalo MDEs provide novel insights into miRNA profiles across different lactation stages, highlighting their physiological role in immunomodulation. The isolation and purification of exosomes from buffalo milk are critical for downstream characterization and miRNA sequencing [21]. In the present study, buffalo MDEs were successfully isolated using differential ultracentrifugation followed by sucrose density gradient centrifugation, exhibiting classic saucer-like morphology and positive biomarkers (CD63, CD81, and TSG101). These findings are consistent with previous reports [22,23], confirming that the isolation strategies effectively reduce interference from high-abundance casein and milk fat globules in complex matrices. Crucially, the structural constancy of these isolated MDEs across distinct lactational matrices establishes a reliable baseline, allowing us to accurately profile the dynamic turnover of their internal miRNA cargo.

Regarding stage-specific characteristics, we first focused on colostrum exosomes, which exhibited a distinct large-particle phenotype with a bimodal distribution, while exosomes from mature milk were considerably smaller. Similar observations of extracellular vesicle sizes in colostrum have been reported for buffalo [24], as well as Holstein and Jersey cows [18]. Colostrum-derived MDEs carry higher levels of immunoglobulins and complement proteins, likely facilitated by molecular crowding effects, which may contribute to an increased hydrodynamic diameter, supporting neonatal passive immunity [8,25]. Due to their gastrointestinal stability, MDEs hold potential as endogenous nanoscale delivery systems [13,26].

For the developing neonatal organism, these colostrum MDEs serve as indispensable vectors for immediate maternal–neonatal communication. It is speculated that the high abundance of immune-related miRNAs in colostrum directly primes mucosal immunity and promotes the maturation of the naive neonatal immune system during the critical first day of life, prior to gut closure [3,27], Notably, the miRNA profile of MDEs shifts significantly during lactation stages. This finding aligns with cross-species studies [28,29], suggesting temporal specificity and evolutionary conservation of MDE miRNA expression.

This dynamic temporal shift in exosomal cargo provides novel mechanistic insights into maternal lactational strategies: exosomal content is not static but is rigorously reprogrammed to match the changing developmental needs of the neonate. The rapid transition from colostrum to mature milk reflects a shift in neonatal needs, from an urgent requirement for immune defense to maintenance of long-term metabolic homeostasis. Furthermore, research has shown that milk exosomes are actively internalized by intestinal epithelial and immune cells, enabling cross-regulation [30], which further underscores their biological significance in early-life programming.

Among their cargo, MDE miRNAs serve as epigenetic messengers that reshape our understanding of dairy nutrition. miRNAs are small non-coding RNAs that regulate target gene expression [9,31]. Among the top 10 highly expressed miRNAs, six belong to the *let-7* family (*let-7a-5p*, *let-7b*, *let-7c*, *let-7f*, *let-7g*, and *let-7i*), making it the most abundant miRNA class in buffalo milk. The *let-7* family comprises 12 gene members encoding 10 mature miRNAs (*let-7a*, *let-7b*, *let-7c*, *let-7d*, *let-7e*, *let-7f*, *let-7g*, *let-7h*, *let-7i*, and *miR-98*), and its members are involved in cell proliferation, apoptosis, signaling, and immune regulation [32]. Previous studies demonstrate that *let-7* inhibition enhances reprogramming efficiency through the Lin41/EGR1 pathway [33], while *let-7c* can activate C/EBP-δ expression to promote macrophage polarization toward the M2 anti-inflammatory phenotype [14,34].

MDEs can regulate metabolic homeostasis and exert therapeutic effects through novel mechanisms [35,36,37]. In this study, buffalo MDEs incubated with macrophages significantly inhibited the transcription of pro-inflammatory factors, including *Ptgs2*, *Mmp9*, and *Tnf*, while upregulating genes associated with cell proliferation. These findings are consistent with observations in yak MDEs [38]. Additionally, milk exosomes have been reported to suppress inflammation by inhibiting the TLR4/NF-κB signaling pathway [39]. Specifically, the concurrent upregulation of *Pik3r3* and downregulation of inflammatory factors supports a model in which buffalo MDEs remodel macrophage phenotypes via a miRNA-Target-PI3K/Akt axis.

While our findings offer preliminary insights into miRNA profiles of buffalo MDEs across the early postpartum transition, several limitations of the present study should be acknowledged. The relatively small sample size may introduce individual biological variation. Exosome characterization would benefit from additional supporting evidence and appropriate negative controls. Furthermore, the findings are based on miRNA profiling and bioinformatic analysis, without direct validation of miRNA-mRNA interactions. The expression levels of crucial DE-miRNAs and their targeted genes require further validation by quantitative real-time PCR (qRT-PCR) to corroborate the sequencing data. Future studies should employ miRNA interference approaches to enable more precise dissection of direct miRNA-mRNA interactions. In additional, systematic in vitro and in vivo experiments in macrophages, including assessments of cell proliferation, viability, apoptosis, fluorescent colocalization of macrophages and MDEs, and intestinal effects in animal models, are warranted to further clarify the immune-regulatory functions of buffalo MDEs.

## 4. Materials and Methods

### 4.1. Animals

Fresh buffalo colostrum and milk samples were obtained from the Guangxi Buffalo Research Institute, Chinese Academy of Agricultural Sciences. A total of six healthy, multiparous lactating buffaloes (Murrah, *n* = 6, 3–4 years of age, second parity, with an average daily milk yield of 20 ± 1 kg/day) were selected for this study. During the periparturient and early lactation periods, all animals were housed in a standardized free-stall barn and fed a total mixed ration twice daily, formulated to meet the nutritional requirements of lactating buffaloes.

Calves were separated from their dams after receiving the initial colostrum. Buffaloes were mechanically milked twice daily. To capture the critical window of neonatal immunological transition [27], milk samples were collected on days 1, 4, and 10 postpartum. The first day secretion is strictly classified as colostrum (BC, day 1) due to its specific immunological cargo. Subsequent samples represent transitional milk (BT, day 4) and mature milk (BM, day 10). Approximately 500 mL of milk per animal was collected into sterile containers and immediately transported to the laboratory at 4 °C. All animal procedures were approved by the Animal Care and Use Committee of Guangxi University (Approval No. SKLCUSA-2023-A04).

### 4.2. Isolation and Purification of MDEs

MDEs were isolated and purified using differential ultracentrifugation combined with sucrose density gradient centrifugation. Milk samples were defatted and clarified by sequential centrifugation at 8000× *g* and 15,000× *g* for 30 min at 4 °C using a high-speed centrifuge equipped with a JA-20 rotor (Beckman Coulter, Brea, CA, USA). The supernatant underwent sequential ultracentrifugation at 50,000× *g* for 1 h and 70,000× *g* for 1 h at 4 °C using an Optima XPN-100 ultracentrifuge (Beckman Coulter). The resulting supernatant was filtered through a 0.45 µm filter and ultracentrifuged at 120,000× *g* for 1 h at 4 °C to pellet the exosomes. The pellet was resuspended in phosphate-buffered saline (PBS), filtered through a 0.22 µm filter, and further purified on a 30% (*w*/*v*) sucrose cushion at 120,000× *g* for 1 h at 4 °C using the Optima XPN-100 ultracentrifuge (Beckman Coulter, Brea, CA, USA) equipped with an SW 32 Ti rotor (Beckman Coulter, Brea, CA, USA) to pellet the exosomes. The pellet was resuspended in PBS, filtered through a 0.22 µm filter, and further purified on a 30% (*w*/*v*) sucrose cushion at 120,000× *g* for 1 h at 4 °C. The exosome-enriched fraction was collected, washed with PBS, and centrifuged at 120,000× *g* for 90 min. Purified MDEs were resuspended in PBS and stored at −80 °C. Crucially, this entire isolation procedure was independently performed for each of the 6 biological replicates per lactation stage, resulting in a total of 18 independent MDE preparations.

### 4.3. Identification and Characterization of MDEs

The morphology of MDEs was visualized using a transmission electron microscope (TEM, Hitachi HT-7700, Tokyo, Japan). Briefly, purified MDEs were placed on a copper grid (Zhongjing Keyi, Beijing, China) for 20 min, stained with 2% uranyl acetate, and air-dried before imaging. Particle size distribution and concentration were determined using a nanoparticle-tracking analyzer (NTA, Zetasizer Nano ZS90, Malvern Panalytical, Malvern, UK). For Western blotting, proteins were separated by SDS-PAGE (Bio-Rad, Laboratories, Hercules, CA, USA) and transferred onto PVDF membranes (Pall Corporation, Port Washington, NY, USA). Membranes were blocked with 5% non-fat milk for 2 h and incubated overnight at 4 °C with primary antibodies against CD63 (Abcam, Cambridge, UK ab216130), CD81 (Abcam, ab109201), TSG101 (Abcam, ab125011), and HSP70 (Abcam, ab181606). After washing, membranes were incubated with HRP-conjugated secondary antibodies (L3012, Signalway Antibody, College Park, MD, USA) for 1 h. Protein bands were visualized using an imaging system (ChemiDoc MP, Bio-Rad, Hercules, CA, USA).

### 4.4. MDEs Incubated with RAW246.7 Cells

RAW264.7 cells were obtained from the Cell Bank of the Chinese Academy of Sciences (**Shanghai, China**). Cells were cultured in DMEM medium (Procell Biotech, Wuhan, China) supplemented with 10% FBS at 37 °C in a humidified atmosphere with 5% CO_2_. Upon reaching 80–90% confluency, RAW264.7 cells were detached for subculture and subsequently incubated with MDEs for 24 h at 37 °C. The harvested cells were isolated for RNA sequencing.

### 4.5. miRNA Sequencing of MDEs and RNA Sequencing of Macrophages

Total RNA was extracted from MDEs using Qiazol Lysis Reagent (Qiagen, Hilden, Germany) and the miRNeasy Mini Kit (Qiagen, Hilden, Germany), following the manufacturer’s instructions. The extracted RNA was resuspended in 50 μL of nuclease-free water, and the integrity and concentration of the total RNA were assessed using an Agilent 2100 Bioanalyzer (Agilent Technologies, Santa Clara, CA, USA). A total of 3 independent small RNA libraries were constructed (*n* = 6 biological replicates for each of the three lactation stages) using the QIAseq miRNA Library Kit (Qiagen, Frederick, MD, USA). To evaluate the transcriptome of macrophages induced by MDEs, RNA libraries were prepared using the mRNA HyperPrep Kit (Roche, Diagnostics, Indianapolis, IN, USA). Libraries were then subjected to high-throughput sequencing on an Illumina platform (Illumina, San Diego, CA, USA).

### 4.6. Bioinformatics Analysis

For bioinformatics analysis, raw reads were processed using to remove adapters and low-quality sequences, followed by assessment. Clean reads were aligned to the Rfam database using to filter non-coding RNAs and mapped to the buffalo genome. miRNA quantification was performed with miRDeep2, applying a threshold of |log_2_(Fold Change)| ≥ 1 (FDR ≤ 0.05). Differential expression analysis was rigorously executed using the R package [40]. This dispersion-aware framework models raw count data based on a negative binomial distribution, incorporating shrinkage estimation for dispersion and fold changes. To control the false discovery rate (FDR), *p*-values for differential expression were adjusted using the Benjamini–Hochberg multiple-testing correction procedure. Target genes were predicted through miRanda software (v3.3a), with a significance threshold of *p* < 0.05. Gene Ontology (GO) and Kyoto Encyclopedia of Genes and Genomes (KEGG) pathway enrichment analyses were conducted for functional annotation. GO terms were calculated using the Benjamini–Hochberg method, KEGG pathway enrichment was assessed via hypergeometric tests (*p* < 0.05).

### 4.7. Data Processing and Analysis

Statistical analyses were performed using GraphPad Prism 9.5 (**GraphPad Software, San Diego, CA, USA**). Data were evaluated using one-way ANOVA followed by the Holm-Šídák test. Results are presented as mean ± standard deviation (mean ± SD), with different letters indicating statistically significant differences (*p* < 0.05).

## 5. Conclusions

This study presents a preliminary analysis of the dynamic changes in miRNA in buffalo MDEs across different lactation stages. The results revealed distinct stage-specific clustering patterns in miRNA expression. Differential expression analysis identified 100 miRNAs that were significantly differentially expressed in colostrum MDEs. Predicted target genes of these DE-miRNA are potentially associated with immune regulation, inflammatory response, and apoptosis pathways. These findings provide new insights into the functional roles of immune-related miRNAs and provide a theoretical foundation for developing immunomodulatory applications of buffalo MDEs. However, several limitations of the present study should be noted, including a small sample size, insufficient exosome characterization and a lack of validation by miRNA interference. Therefore, further in vitro and in vivo studies in macrophages are warranted to clarify the immune-regulatory functions of buffalo MDEs.

## Figures and Tables

**Table 1:** Summary of sequencing data quality and mapping statistics.

Sample	Raw Reads	Clean Reads	Q30 (%)	GC Content (%)	Total Reads	Mapped Reads
BC	13,855,157	13,758,228	97.70	45.00	233,985	9,529,106
BT	15,444,148	15,347,736	97.63	48.00	591,610	5,712,656
BM	13,082,494	13,006,482	97.33	47.50	361,773	11,642,449

## Data Availability

The original data presented in this study are included in the article and Supplementary Material; further inquiries can be directed to the corresponding author.

## Supplementary Materials

The following supporting information can be downloaded at: https://www.mdpi.com/article/10.3390/molecules31081332/s1, Tables S1 and S2: The top 20 most abundant miRNAs in buffalo MDEs across different lactation stages; miRNAs specifically expressed in buffalo colostrum-derived exosomes. Table S3: Significant GO terms and KEGG pathways enriched by predicted target genes of DE-miRNAs. Table S4: Raw count matrix and size-factor normalized expression values.
