# Supplementary material for: Comprehensive Analysis of Exosomal microRNAs in Buffalo Milk Across the Early Postpartum Transition
Source: Molecules. 2026 Apr 18;31(8):1332. doi: 10.3390/molecules31081332 (PMC13119057; doi:10.3390/molecules31081332)
Supplement: Supplementary file 1 [file molecules-31-01332-s001.zip › Supplementary Table 1&2.pdf]

Table S1. The top 20 most abundant miRNAs in buffalo MDEs across different lactation stages

No.	miRNA ID	Sequence (5'-3')	Colostrum (BC)	Transitional (BT)	Mature (BM)	Family
1	bta-let-7a-5p	UGAGGUAGUAGGUUGUAUAGUU	181,779.2	197,822.4	204,073.6	let-7
2	bta-let-7b	UGAGGUAGUAGGUUGUGUGGUU	145,650.6	175,686.5	170,974.3	let-7
3	bta-let-7f	UGAGGUAGUAGAUUGUAUAGUU	49,250.6	34,806.9	36,752.6	let-7
4	bta-let-7g	UGAGGUAGUAGUUUGUACAGUU	27,009.3	16,370.7	14,925.7	let-7
5	bta-let-7c	UGAGGUAGUAGGUUGUAUGGUU	16,887.9	18,033.6	15,194.0	let-7
6	bta-miR-26a	UUCAAGUAAUCCAGGAUAGGCU	18,353.2	12,299.5	15,054.7	miR-26
7	bta-miR-30a-5p	UGUAAACAUCCUCGACUGGAAG	14,326.3	11,661.5	15,348.0	miR-30
8	bta-miR-16a	UAGCAGCACGUAAAUAUUGGCG	11,627.8	9,153.8	9,847.1	miR-16
9	bta-miR-191	CAACGGAAUCCCAAAAGCAGCUG	7,962.7	7,272.3	8,137.2	miR-191
10	bta-miR-200c	UAAUACUGCCGGGUAAUGAUGGA	5,482.8	6,855.9	8,452.2	miR-200
11	bta-miR-423-5p	UGAGGGGCAGAGAGCGAGACUUU	3,845.3	8,390.4	8,209.2	miR-423
12	bta-miR-151-5p	UCGAGGAGCUCACAGUCUAGU	4,860.2	6,021.8	6,353.4	miR-151
13	bta-let-7i	UGAGGUAGUAGUUUGUGCUGUU	8,092.2	4,974.5	4,006.0	let-7
14	bta-miR-320a	AAAAGCUGGGUUGAGAGGGCGA	3,408.0	5,875.3	6,281.6	miR-320
15	bta-let-7e	UGAGGUAGGAGGUUGUAUAGU	3,730.3	3,315.7	2,735.8	let-7
16	bta-miR-30d	UGUAAACAUCCCCGACUGGAAG	2,699.0	3,602.8	3,401.4	miR-30
17	bta-miR-26b	UUCAAGUAAUUCAGGAUAGGUU	2,823.6	2,397.1	2,844.4	miR-26
18	bta-let-7d	AGAGGUAGUAGGUUGCAUAGUU	3,099.5	2,187.5	1,987.5	let-7
19	bta-miR-93	CAAAGUGCUGUUCGUGCAGGUA	2,711.9	1,776.6	1,252.7	miR-93
20	bta-miR-98	UGAGGUAGUAAGUUGUAUUGUU	2,049.6	1,667.8	1,681.3	let-7*

Table S2. miRNAs specifically expressed in buffalo colostrum-derived exosomes

miRNA	Length	Sequence
bbu-miR-10162-5p	23	UGGACAGGCCAAGCCGCUGUGCA
bbu-miR-10175-5p	20	UGGAGAGAACAGGUGGCUUU
bbu-miR-10181-5p	21	UGUGGGAAGGAGCUGUGCUGG
bbu-miR-10182-5p	24	CAGUCCGGUCCCGCGGUGUCUCCG
bbu-miR-11977	20	UGUCUCAGUUCAGCAGGAAG
bbu-miR-12010	23	UGGAGGCUGGGAGUGCAUGAGCU
bbu-miR-138	23	AGCUGGUGUUGUGAAUCAGGCCG
bbu-miR-1388-3p	22	AUCUCAGGUUUGUCAGCCCGCA
bbu-miR-1388-5p	21	AGGACUGUCCAACCUGAGAAU
bbu-miR-153	22	UUGCAUAGUCACAAAAGUGAUC
bbu-miR-1842	21	UGGCUCUGUGAGGUCGGCUCA
bbu-miR-191b	22	GAACGAAAUCCAAGCGCAGCUG
bbu-miR-193a-3p	22	AACUGGCCUACAAAGUCCCAGU
bbu-miR-199a-3p	22	ACAGUAGUCUGCACAUUGGUUA
bbu-miR-199c	20	UACAGUAGUCUGCACAUUGG
bbu-miR-19a	23	UGUGCAAAUCUAUGCAAAACUGA
bbu-miR-218	22	UUGUGCUUGAUCUAACCAUGUG
bbu-miR-222	21	AGCUACAUCUGGCUACUGGGU
bbu-miR-2284aa	21	AAAAAAGUUUGUUUGGGUUUU
bbu-miR-2284d	22	AAAAAGUUCGUUAGGGUUUUUC
bbu-miR-2284l	20	AAAAGUUGGUUCGGGUUUUU
bbu-miR-2285al	21	ACAAAGUUUGUUCGGGUUUUA
bbu-miR-2285bt	22	AAAAAGUUCAUUUGGGUUGUCU
bbu-miR-2285k	21	AAAACCGGAAUGAACUUUUUG
bbu-miR-2354	21	UAGUAGGUUGUGUUGUUUAGU
bbu-miR-2370-3p	21	UAAGCAACUUUCCUUUCUCCA
bbu-miR-2419-3p	22	CAGGCGGGUGCUAAUACGAUCA
bbu-miR-2475	23	UGAAGUGUGUGAAACUGCAGCGG
bbu-miR-301a	25	CAGUGCAAUAGUAUUGUCAAAGCAU
bbu-miR-34c	22	AGGCAGUGUAGUUAGCUGAUUG
bbu-miR-362-3p	21	AACACACCUAUUCAAGGAUUC
bbu-miR-376a	21	AUCAUAGAGGAAAAUCCACGU
bbu-miR-376e	22	AACAUAGAGGAAAAUCCACAUU
bbu-miR-378d	22	CUGGACUUGGAGUCAGAAGACC
bbu-miR-383	22	AGAUCAGAAGGUGAUUGUGGCU
bbu-miR-4449	22	CGUCCCGGGGCCGCUCGAGGCA
bbu-miR-450b	22	UUUUGCAAUAUGUUCCUGAAUA
bbu-miR-502a	22	AAUGCACCUGGGCAAGGAUUCA
bbu-miR-582	23	UUACAGUUGUUCAACCAGUUACU
bbu-miR-6501	23	CCAGGGCAGCCUGUGGUAACAGU
bbu-miR-6518	22	UCACGGAGAGGAGAAACUGCAC
bbu-miR-8549	17	GAGGUAGUAGGUGGUAU
bbu-miR-874	22	CUGCCCUGGCCCGAGGGACCGA
bbu-miR-9-3p	17	AUAAAGCUAGAUAACCG
